# Mixed-Norm Regularization for Brain Decoding

**DOI:** 10.1155/2014/317056

**Published:** 2014-04-17

**Authors:** R. Flamary, N. Jrad, R. Phlypo, M. Congedo, A. Rakotomamonjy

**Affiliations:** ^1^Laboratoire Lagrange, UMR7293, Université de Nice, 00006 Nice, France; ^2^Gipsa Lab, Domaine Universitaire BP 46, 38402 Saint Martin d'Hères, France; ^3^LITIS, EA 4108-INSA, Université de Rouen, 76000 Rouen, France

## Abstract

This work investigates the use of mixed-norm regularization for sensor selection in event-related potential (ERP) based brain-computer interfaces (BCI). The classification problem is cast as a discriminative optimization framework where sensor selection is induced through the use of mixed-norms. This framework is extended to the multitask learning situation where several similar classification tasks related to different subjects are learned simultaneously. In this case, multitask learning helps in leveraging data scarcity issue yielding to more robust classifiers. For this purpose, we have introduced a regularizer that induces both sensor selection and classifier similarities. The different regularization approaches are compared on three ERP datasets showing the interest of mixed-norm regularization in terms of sensor selection. The multitask approaches are evaluated when a small number of learning examples are available yielding to significant performance improvements especially for subjects performing poorly.

## 1. Introduction

Brain computer interfaces (BCI) are systems that help disabled people communicate with their environment through the use of brain signals [1]. At the present time, one of the most prominent BCI is based on electroencephalography (EEG) because of its low-cost, portability, and its noninvasiveness. Among EEG based BCI, a paradigm of interest is the one based on event-related potentials (ERP) which are responses of the brain to some external stimuli. In this context, the innermost part of a BCI is the pattern recognition stage which has to correctly recognize presence of these ERPs. However, EEG signals are blurred due to the diffusion of the skull and the skin [2]. Furthermore, EEG recordings are highly contaminated by noise of biological, instrumental, and environmental origins. For addressing these issues, advanced signal processing and machine learning techniques have been employed to learn ERP patterns from training EEG signals leading to robust systems able to recognize the presence of these events [3–8]. Note that while some ERPs are used for generating BCI commands, some others can be used for improving BCI efficiency. Indeed, recent studies have also tried to develop algorithms for automated recognition of error-related potentials [9]. These potentials are responses elicited when a subject commits an error in a BCI task or observes an error [10, 11] and thus they can help in correcting errors or in providing feedbacks to BCI users.

In this context of automated recognition of event-related potentials for BCI systems, reducing the number of EEG sensors is of primary importance since it reduces the implementation cost of the BCI by minimizing the number of EEG sensor and speeding up experimental setup and calibration time. For this purpose, some studies have proposed to choose relevant sensors according to prior knowledge of brain functions. For instance, sensors located above the motor cortex region are preferred for motor imagery tasks, while for visual event-related potential (ERP), sensors located on the visual cortex are favored [12]. Recent works have focused on automatic sensor selection adapted to the specificity of a subject [4, 13–17]. For instance, Rakotomamonjy and Guigue [18] performed a recursive backward sensor selection using cross-validation classification performances as an elimination criterion. Another approach for exploring subset sensors has been proposed by [15]; it consists in using a genetic algorithm for sensor selection coupled with artificial neural networks for prediction. Those methods have been proven efficient but computationally demanding. A quicker way is to estimate the relevance of the sensors in terms of signal to noise ratio (SNR) [4] and to keep the most relevant ones. Note that this approach does not optimize a discrimination criterion, although the final aim is a classification task. Recently, van Gerven et al. [19] proposed a graceful approach for embedding sensor selection into a discriminative framework. They performed sensor selection and learn a decision function by solving a unique optimization problem. In their framework, a logistic regression classifier is learned and the group-lasso regularization, also known as *ℓ*_1_ − *ℓ*_2_ mixed-norm, is used to promote sensor selection. They have also investigated the use of this groupwise regularization for frequency band selection and their applications to transfer learning. The same idea has been explored by Tomioka and Müller [20] which also considered groupwise regularization for classifying EEG signals. In this work, we go beyond these studies by providing an in-depth study of the use of mixed-norms for sensor selection in a single subject setting and by discussing the utility of mixed-norms when learning decision functions for multiple subjects simultaneously.

Our first contribution addresses the problem of robust sensor selection embedded into a discriminative framework. We broaden the analysis of van Gerven et al. [19] by considering regularizers whose forms are *ℓ*_1_ − *ℓ*_*q*_ mixed-norms, with (1 ≤ *q* ≤ 2), as well as adaptive mixed-norms, so as to promote sparsity among group of features or sensors. In addition to providing a sparse and accurate sensor selection, mixed-norm regularization has several advantages. First, sensor selection is cast into an elegant discriminative framework, using for instance a large margin paradigm, which does not require any additional hyperparameter to be optimized. Secondly, since sensor selection is jointly learned with the classifier by optimizing an “all-in-one” problem, selected sensors are directed to the goal of discriminating relevant EEG patterns. Hence, mixed-norm regularization helps locating sensors which are relevant for an optimal classification performance.

A common drawback of all the aforementioned sensor selection techniques is that selected set of sensors may vary, more or less substantially, from subject to subject. This variability is due partly to subject specific differences and partly to acquisition noise and limited number of training examples. In such a case, selecting a robust subset of sensors may become a complex problem. Addressing this issue is the point of our second contribution. We propose a multitask learning (MTL) framework that helps in learning robust classifiers able to cope with the scarcity of learning examples. MTL is one way of achieving inductive transfer between tasks. The goal of inductive transfer is to leverage additional sources of information to improve the performance of learning on the current task. The main hypothesis underlying MTL is that tasks are related in some ways. In most cases, this relatedness is translated into a prior knowledge, for example, a regularization term, that a machine learning algorithm can take advantage of. For instance, regularization terms may promote similarity between all the tasks [21] or enforce classifier parameters to lie in a low dimensional linear subspace [22] or to jointly select the relevant features [23]. MTL has been proven efficient for motor imagery in [24] where several classifiers were learned simultaneously from several BCI subject datasets. Our second contribution is thus focused on the problem of performing sensor selection and learning robust classifiers through the use of an MTL mixed-norm regularization framework. We propose a novel regularizer promoting sensor selection and similarity between classifiers. By doing so, our goal is then to yield sensor selection and robust classifiers that are able to overcome the data scarcity problem by sharing information between the different classifiers to be learned.

The paper is organized as follows. The first part of the paper presents the discriminative framework and the different regularization terms we have considered for channel selection and multitask learning. The second part is devoted to the description of the datasets, the preprocessing steps applied to each of them, and the results achieved in terms of performances and sensor selection. In order to promote reproducible research, the code needed for generating the results in this paper is available on the author's website (URL: http://remi.flamary.com/soft/soft-gsvm.html.).

## 2. Learning Framework

In this section, we introduce our mixed-norm regularization framework that can be used to perform sensor selection in a single task or in a transfer learning setting.

### 2.1. Channel Selection in a Single Task Learning Setting

Typically in BCI problems, one wants to learn a classifier that is able to predict the class of some EEG trials, from a set of learning examples. We denoted as {**x**_*i*_, *y*_*i*_}_*i*∈{1,…,*n*}_ the learning set such that **x**_*i*_ ∈ ℝ^*d*^ is a trial and *y*_*i*_ ∈ {−1,1} is its corresponding class, usually related to the absence or presence of an event-related potential. In most cases, a trial **x**_*i*_ is extracted from a multidimensional signal and thus is characterized by *r* features for each of the *p* sensors, leading to a dimensionality *d* = *r* × *p*. Our aim is to learn, for a single subject, a linear classifier *f* that will predict the class of a trial **x** ∈ ℝ^*d*^, by looking at the sign of the function *f*(·) defined as

(1) $$\begin{array}{c} f\left(x\right)=x^{T}w+b \end{array}$$

with **w** ∈ ℝ^*d*^ the normal vector to the separating hyperplane and *b* ∈ ℝ a bias term. Parameters of this function are learned by solving the optimization problem:

(2) $$\begin{array}{c} \underset{w,b}{\min}\overset{n}{\underset{i}{\sum }}L_{o}\left(y_{i},x_{i}^{T}w+b\right)‍\;+\lambda \Omega \left(w\right), \end{array}$$

where *L*_*o*_ is a loss function that measures the discrepancy between actual and predicted labels, *Ω*(·) is a regularization term that expresses some prior knowledge about the learning problem, and *λ* is a parameter that balances both terms. In this work, we choose *L*_*o*_ to be the squared hinge loss $L_{o}(y,\hat{y})=\max{\left(0,1-y\hat{y}\right)}^{2}$, thus promoting a large margin classifier.

#### 2.1.1. Regularization Terms

We now discuss different regularization terms that may be used for single task learning along with their significances in terms of channel selection.

*ℓ*_2_ *Norm*. The first regularization term that comes to mind is the standard squared *ℓ*_2_ norm regularization:

(3) $$\begin{array}{c} \Omega_{2}\left(w\right)=\frac{1}{2}{\left|\left|w\right|\right|}_{2}^{2}, \end{array}$$

where ||·||_2_ is the Euclidean norm. This is the common regularization term used for SVMs and it will be considered in our experiments as the baseline approach. Intuitively, this regularizer tends to downweigh the amplitude of each component of **w** leading to a better control of the margin width of our large-margin classifier and thus it helps in reducing overfitting. 

*ℓ*_1_ *Norm.* When only few of the features are discriminative for a classification task, a common way to select the relevant ones is to use an *ℓ*_1_ norm of the form

(4) $$\begin{array}{c} \Omega_{1}\left(w\right)=\overset{d}{\underset{i=1}{\sum }}\left|w_{i}\right|‍\; \end{array}$$

as a regularizer [25]. Owing to its mathematical properties (nondifferentiability at 0), unlike the *ℓ*_2_ norm, this regularization term promotes sparsity, which means that at optimality of problem (2), some components of **w** are exactly 0. In a Bayesian framework, the *ℓ*_1_ norm is related to the use of prior on **w** that forces its component to vanish [19]. This is typically obtained by means of Laplacian prior over the weight. However, *ℓ*_1_ norm ignores the structure of the features (which may be grouped by sensors) since each component of *w* is considered independently to the others. As such, this norm precludes grouped feature selection and allows only for feature selection.

**Figure 1:**
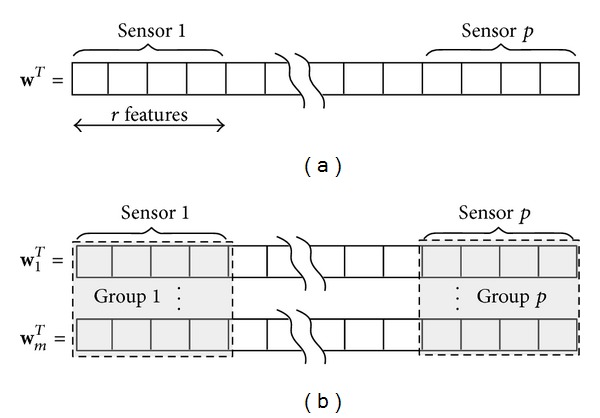
Examples of feature grouping for (a) single task and (b) multiple task learning.

*ℓ*_1_ − *ℓ*_*q*_ *Mixed-Norm.* A way to take into account the fact that features are structured is to use a mixed-norm that will group them and regularize them together. Here, we consider mixed-norm of the form

(5) $$\begin{array}{c} \Omega_{1-q}\left(w\right)=\underset{g\in 𝒢}{\sum }{\left|\left|w_{g}\right|\right|}_{q}‍\; \end{array}$$

with 1 ≤ *q* ≤ 2 and *𝒢* being a partition of the set {1,…, *d*}. Intuitively, this *ℓ*_1_ − *ℓ*_*q*_ mixed-norm can be interpreted as an *ℓ*_1_ norm applied to the vector containing the *ℓ*_*q*_ norm of each group of features. It promotes sparsity on each **w**_*g*_ norm and consequently on the **w**_*g*_ components as well. For our BCI problem, a natural choice for *𝒢* is to group the features by sensors yielding thus to *p* groups (one per sensor) of *r* features as reported in Figure 1. Note that unlike the *ℓ*_1_ − *ℓ*_2_ norm as used by van Gerven et al. [19] and Tomioka and Müller [20], the use of an inner *ℓ*_*q*_ norm leads to more flexibility as it spans from the *ℓ*_1_ − *ℓ*_1_ (equivalent to the *ℓ*_1_-norm and leading thus to unstructured feature selection) to the *ℓ*_1_ − *ℓ*_2_ which strongly ties together the components of a group. Examples of the use of *ℓ*_*q*_ norm and mixed-norm regularizations in other biomedical contexts can be found for instance in [26, 27].

*Adaptive* *ℓ*_1_ − *ℓ*_*q*_. The *ℓ*_1_ and *ℓ*_1_ − *ℓ*_*q*_ norms described above are well known to lead to grouped feature selection. However, they are also known to lead to poor statistical properties (at least when used with a square loss function) [28]. For instance, they are known to have consistency issue in the sense that, even with an arbitrarily large number of training examples, these norms may be unable to select the true subset of features. In practice, this means that when used in (2), the optimal weight vector **w** will tend to overestimate the number of relevant sensors. These issues can be addressed by considering an adaptive *ℓ*_1_ − *ℓ*_*q*_ mixed-norm of the form [28, 29]

(6) $$\begin{array}{c} \Omega_{a:1-q}\left(w\right)=\underset{g\in 𝒢}{\sum }\beta_{g}{\left|\left|w_{g}\right|\right|}_{q}‍, \end{array}$$

where the weights *β*_*g*_ are selected so as to enhance the sparsity pattern of **w**. In our experiments, we obtain them by first solving the *ℓ*_1_ − *ℓ*_*q*_ problem with *β*_*g*_ = 1, which outputs an optimal parameter w*, and by finally defining *β*_*g*_ = 1/||**w**_g_* | |_*q*_. Then, solving the weighted *ℓ*_1_ − *ℓ*_*q*_ problem yields an optimal solution with increased sparsity pattern compared to w* since the *β*_*g*_ augments the penalization of groups with norm ||**w**_g_*||_*q*_ smaller than 1.

#### 2.1.2. Algorithms

Let us now discuss how problem (2) is solved when one of these regularizers is in play.

Using the *ℓ*_2_ norm regularization makes the problem differentiable. Hence a first- or second-order descent based algorithm can be considered [30].

Because the other regularizers are not differentiable, we have deployed an algorithm [31] tailored for minimizing objective function of the form *f*_1_(**w**) + *f*_2_(**w**) with *f*_1_ a smooth and differentiable convex function with Lipschitz constant *L* and *f*_2_ a continuous and convex nondifferentiable function having a simple proximal operator, that is, a closed-form or an easy-to-compute solution of the problem

(7) $$\begin{array}{c} {\text{prox}}_{f_{2}}\left(v\right):=\underset{u}{\text{argmin}}\frac{1}{2}{||v-u||}_{2}^{2}+f_{2}\left(u\right). \end{array}$$

Such an algorithm, known as forward-backward splitting [31], is simply based on the following iterative approach:

(8) $$\begin{array}{c} w^{k+1}=\text{pro}{\text{x}}_{(1/\gamma )f_{2}}\left(w^{k}-\gamma \nabla_{w}f_{1}\left(w^{k}\right)\right) \end{array}$$

with *γ* being a stepsize in the gradient descent. This algorithm can be easily derived by considering, instead of directly minimizing *f*_1_(**w**) + *f*_2_(**w**), an iterative scheme which at each iteration replaces *f*_1_ with a quadratic approximation of *f*_1_(·) in the neighborhood of **w** ^*k*^. Hence, **w** ^*k*+1^ is the minimizer of

(9) $$\begin{array}{c} f_{1}\left(w^{k}\right)+\langle \nabla_{w}f_{1}\left(w^{k}\right),w-w^{k}\rangle +\frac{\gamma }{2}{||w-w^{k}||}_{2}^{2}+f_{2}\left(w\right) \end{array}$$

whose closed-form is given in (8). This algorithm is known to converge towards a minimizer of *f*_1_(**w**) + *f*_2_(**w**) under some weak conditions on the stepsize [31], which is satisfied by choosing for instance *γ* = 1/*L*. We can note that the algorithm defined in (8) has the same flavor as a projected gradient algorithm which first takes a gradient step and then “projects” back the solution owing to the proximal operator. More details can also be found in [32].

For our problem (2), we choose *f*_1_(**w**) to be the squared hinge loss and *f*_2_(**w**) the nonsmooth regularizer. The square hinge loss is indeed gradient Lipschitz with a constant *L* being 2∑_*i*=1_||**x**_*i*_||_2_^2^. Proof of this statement is available in Appendix A. Proximal operators of the *ℓ*_1_ and the *ℓ*_1_ − *ℓ*_2_ regularization term can be easily shown to be the soft-thresholding and the block-soft thresholding operator [25]. The general *ℓ*_1_ − *ℓ*_*q*_ norm does not admit a closed-form solution, but its proximal operator can be simply computed by means of an iterative algorithm [23]. More details on these proximal operators are also available in Appendix C.

### 2.2. Channel Selection and Transfer Learning in Multiple Task Setting

We now address the problem of channel selection in cases where training examples for several subjects are at our disposal. We have claimed that in such a situation, it would be beneficial to learn the decision functions related to all subjects simultaneously, while inducing selected channels to be alike for all subjects, as well as inducing decision function parameters to be related in some sense. These two hypotheses make reasonable sense since brain regions related to the appearance of a given ERP are expected to be somewhat location-invariant across subjects. For solving this problem, we apply a machine learning paradigm, known as multitask learning, where in our case, each task is related to the decision function of a given subject and where the regularizer should reflect the above-described prior knowledge on the problem. Given *m* subjects, the resulting optimization problem boils down to be

(10) $$\begin{array}{c} \underset{W,b}{\min}\overset{m}{\underset{t}{\sum }}\overset{n_{t}}{\underset{i=1}{\sum }}L\left(y_{i,t},x_{i,t}^{T}w_{t}+b_{t}\right)‍‍+\Omega_{\text{mtl}}\left(W\right) \end{array}$$

with {**x**_*i*,*t*_, *y*_*i*,*t*_}_*i*∈{1,…,*n*_*t*_}_ being the training examples related to each task, *t* ∈ 1,…, *m*, (**w**_*t*_, **b**_*t*_) being the classifier parameters for task *t*, and **W** = [**w**_1_,…, **w**_*m*_] ∈ ℝ^*d*×*m*^ being a matrix concatenating all vectors {**w**_*t*_}. Note that the multitask learning framework applied to single EEG trial classification has already been investigated by van Gerven et al. [19]. The main contribution we bring compared to their works is the use of regularizer that explicitly induces all subject classifiers to be similar to an average one, in addition to a regularizer that enforces selected channels to be the same for all subjects. The intuition behind this point is that we believe that since the classification tasks we are dealing with are similar for all subjects and all related to the same BCI paradigm, selected channels and classifier parameters should not differ that much from subject to subject. We also think that inducing task parameters to be similar may be more important than enforcing selected channels to be similar when the number of training examples is small since it helps in reducing overfitting. For this purpose, we have proposed a novel regularization term of the form

(11) $$\begin{array}{c} \Omega_{\text{mtl}}\left(W\right)=\lambda_{r}\underset{g\in {𝒢}^{\prime }}{\sum }{\left|\left|W_{g}\right|\right|}_{2}‍+\lambda_{s}\overset{m}{\underset{t=1}{\sum }}{\left|\left|w_{t}-\hat{w}\right|\right|}_{2}^{2}‍, \end{array}$$

where $\hat{w}=\left(1/m\right)\sum_{t}w_{t}$ is the average classifier across tasks and *𝒢*′ contains nonoverlapping groups of components from matrix **W**. The first term in (11) is a mixed-norm term that promotes group regularization. In this work, we defined groups in *𝒢*′ based on the sensors, which means that all the features across subject related to a given sensor are in the same group *g*, leading to *p* groups of *r* × *m* feature, as depicted in Figure 1. The second term is a similarity promoting term as introduced in Evgeniou and Pontil [21]. It can be interpreted as a term enforcing the minimization of the classifier's parameter variance. In other words, it promotes classifiers to be similar to the average one, and it helps improving performances when the number of learning examples for each task is limited, by reducing overfitting. Note that *λ*_*r*_ and *λ*_*s*_, respectively, control the sparsity induced by the first term and the similarity induced by the second one. Hence, when setting *λ*_*s*_ = 0, the regularizer given in (11) boils down to be similar to the one used by van Gerven et al. [19]. Note that in practice *λ*_*r*_ and *λ*_*s*_ are selected by means of a nested cross-validation which aims at classification accuracy. Thus, it may occur that classifier similarity is preferred over sensor selection leading to robust classifiers which still use most of the sensors.

Similar to the single task optimization framework given in (2), the objective function for problem (10) can be expressed as a sum of gradient Lipschitz continuous term $f_{1}(W)=\sum_{t,i}^{m,n}L(\cdot )+\lambda_{s}\sum_{t=1}^{m}||w_{t}-\hat{w}|{|}_{2}^{2}$ and a nondifferentiable term *f*_2_(**W**) = *λ*_*r*_∑_*g*∈*𝒢*′_ | |**W**_*g*_ | |_2_ having a closed-form proximal operator (see Appendix B). Hence, we have again considered a forward-backward splitting algorithm whose iterates are given in (8).

## 3. Numerical Experiments

We now present how these novel approaches perform on different BCI problems. Before delving into the details of the results, we introduce the simulated and real datasets.

### 3.1. Experimental Data

We have first evaluated the proposed approaches on a simple simulated P300 dataset generated as follows. A P300 wave is extracted using the grand average of a single subject data from the EPFL dataset described in the following. We generate 11000 simulated examples with 8 discriminative channels containing the P300 out of 16 channels for positive examples. A Gaussian noise of standard deviation 0.2 is added to all signals making the dataset more realistic. 1000 of these examples have been used for training.

The first real P300 dataset we used is the EPFL dataset, based on eight subjects performing P300 related tasks [33]. The subjects were asked to focus on one of the 3 × 2 = 6 images on the screen while one of the images is flashed at random. The EEG signals were acquired from 32 channels, sampled at 1024 Hz, and 4 recording sessions per subject have been realized. Signals are preprocessed exactly according to the steps described in [33]: a [1,8] Hz bandpass Butterworth filter of order 3 is applied to all signals followed by a downsampling. Hence, for each trial (training example), we have 8 time-sample features per channel corresponding to a 1000 ms time-window after stimulus, which leads to 256 features for all channels (32 × 8 = 256 features). Overall, the training set of a given subject is composed of about 3000 trials.

Another P300 dataset, recorded by the Neuroimaging Laboratory of Universidad Autónoma Metropolitana (UAM, Mexico) [34], has also been utilized. The data have been obtained from 30 subjects performing P300 spelling tasks on a 6 × 6 virtual keyboard. Signals are recorded over 10 channels leading thus to a very challenging dataset for sensor selection, as there are just few sensors left to select. For this dataset, we only use the first 3 sessions in order to have the same number of trials for all subjects (≈4000 samples). The EEG signals have been preprocessed according to the following steps: a [2,20] Hz Chebyshev bandpass filter of order 5 is first applied followed by a decimation, resulting in a poststimulus time-window of 31 samples per channels. Hence, each trial is composed of 310 (10 × 31) features.

We have also studied the effectiveness of our methods on an error-related potential (ErrP) dataset that has been recorded in the GIPSA Lab. The subjects were asked to memorize the position of 2 to 9 digits and to remind the position of one of these digits; operation has been repeated 72 times for each subject. The signal following the visualization of the result (correct/error on the memorized position) was recorded from 31 electrodes and sampled at 512 Hz. Similar to Jrad et al. [17], a [1,10] Hz Butterworth filter of order 4 and a downsampling has been applied to all channel signals. Finally, a time window of 1000 ms is considered as a trial (training example) with a dimensionality of 16 × 31 = 496.

### 3.2. Evaluation Criterion, Methods, and Experimental Protocol

We have compared several regularizers that induce feature/channel selection embedded in the learning algorithm, in a single subject learning setting as defined in (2). The performance measure commonly used in BCI competitions [3] is the area under the Roc curve (AUC). This measure is an estimate of the probability for a positive class to have a higher score than a negative class. It makes particularly sense to use AUC when evaluating a P300 speller as the letter in the keyboard is usually chosen by comparing score returned by the classifier for every column or line. In addition, AUC does not depend on the proportion of positive/negative examples in the data which makes it more robust than classification error rate. Our baseline algorithm is an SVM, which uses an *ℓ*_2_ regularizer and thus does not perform any selection. Using an *ℓ*_1_ regularizer yields a classifier which embeds feature selection, denoted as SVM-1 in the sequel. Three mixed-norm regularizers inducing sensor selection have also been considered: an *ℓ*_1_ − *ℓ*_2_ denoted as GSVM-2, and *ℓ*_1_ − *ℓ*_*q*_ referred as GSVM-q, with *q* being selected in the set {1,1.2,…, 1.8,2} by a nested cross-validation stage, and adaptive *ℓ*_1_ − *ℓ*_*q*_ norm, with *q* = 2 denoted as GSVM-a.

For the multitask learning setting, two MTL methods were compared to two baseline approaches which use all features, namely, a method that treats each tasks separately by learning one SVM per task (SVM) and a method denoted as SVM-Full, which on the contrary learns a unique SVM from all subject datasets. The two MTL methods are, respectively, a MTL as described in (10), denoted as MGSVM-2s and the same MTL but without similarity promoting regularization term, which actually means that we set *λ*_*s*_ = 0, indicated as MGSVM-2. For these approaches, performances are evaluated as the average AUC of the decision functions over all the subjects.

The experimental setup is described in the following. For each subject, the dataset is randomly split into a training set of *n* = 1000 trials and a test set containing the rest of the trials. The regularization parameter *λ* has been selected from a log-spaced grid ([10^−3^, 10^1^]) according to a nested 3-fold cross-validation step on the training set. When necessary, the selection of *q* is also included in this CV procedure. Finally, the selected value of *λ* is used to learn a classifier on the training examples and performances are evaluated on the independent test set. We run this procedure 10 times for every subject and report average performances. A Wilcoxon signed-rank test, which takes ties into account, is used to evaluate the statistical difference of the mean performances of all methods compared to the baseline SVM. We believe that such a test is more appropriate for comparing methods than merely looking at the standard deviation due to the high intersubject variability in BCI problems.

### 3.3. Results and Discussions

We now present the results we achieved on the above-described datasets.

#### 3.3.1. Simulated Dataset

Average (over 10 runs) performance of the different regularizers on the simulated dataset is reported in Table 1 through AUC, sensor selection rate, and *F*-measure. This latter criterion measures the relevance of the selected channels compared to the true relevant ones. F-measure is formally defined as

(12) $$\begin{array}{c} F\text{-measure}=2\frac{\left|𝒞\cap {𝒞}^{*}\right|}{\left|{𝒞}^{*}\right|+\left|𝒞\right|}, \end{array}$$

where *𝒞* and 𝒞* are, respectively, the set of selected channels and true relevant channels and |·| here denotes the cardinality of a set. Note that if the selected channels are all the relevant ones, then the *F*-measure is equal to one. Most of the approaches provide similar AUC performances. We can although highlight that group-regularization approaches (GSVM-2, GSVM-p, GSVM-a) drastically reduce the number of selected channels since only 62% and 45% of the sensors are selected. A clear advantage goes to the adaptive regularization that is both sparser and is more capable of retrieving the true relevant channels.

#### 3.3.2. P300 Datasets

Results for these datasets are reported in Table 2. For the EPFL dataset, all methods achieve performances that are not statistically different. However, we note that GSVM-2 leads to sensor selection (80% of sensor selected) while GSVM-a yields to classifiers that, on average, use 26% of the sensors at the cost of a slight loss in performances (1.5% AUC).

Results for the UAM dataset follow the same trend in terms of sensor selection but we also observe that the mixed-norm regularizers yield to increased performances. GSVM-2 performs statistically better than SVM although most of the sensors (9 out of 10) have been kept in the model. This shows that even if few channels have been removed, the group-regularization improves performances by bringing sensor prior knowledge to the problem. We also notice that GSVM-a performance is statistically equivalent to the baseline SVM one while using only half of the sensors and GSVM-p consistently gives similar results to GSVM-2.

To summarize, concerning the performances of the different mixed-norm regularization, we outline that on one hand, GSVM-2 is at worst equivalent to the baseline SVM while achieving sensor selection and on the other hand GSVM-a yields to a substantial channel selection at the expense of a slight loss of performances.

**Figure 2:**
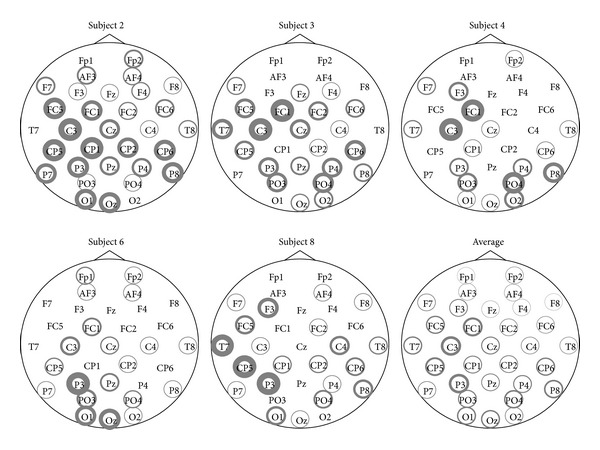
Selected sensors for the EPFL dataset. The line width of the circle is proportional to the number of times the sensor is selected for different splits. No circle means that the sensor has never been selected.

**Figure 3:**
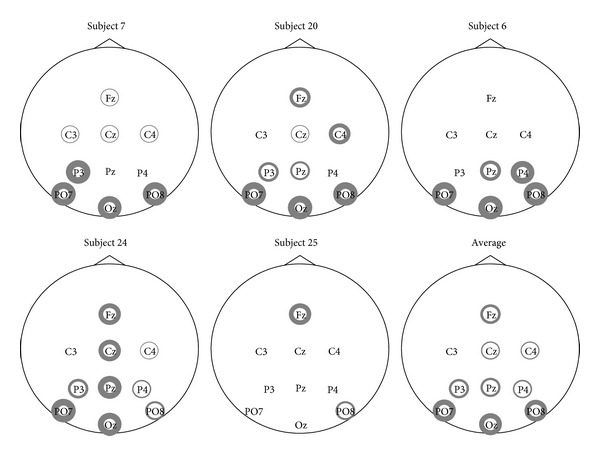
Selected sensors for the UAM dataset. The line width of the circle is proportional to the number of times the sensor is selected for different splits. No circle means that the sensor has never been selected.

A visualization of the electrodes selected by GSVM-a can be seen in Figure 2 for the EPFL dataset and in Figure 3 for the UAM dataset. Interestingly, we observe that for the EPFL dataset, the selected channels are highly dependent on the subject. The most recurring ones are the following: FC1 C3 T7 CP5 P3 PO3 PO4 Pz and the electrodes located above visual cortex O1, Oz, and O2. We see sensors from the occipital area that are known to be relevant [12] for P300 recognition, but sensors such as T7 and C3, from other brain regions, are also frequently selected. These results are however consistent with those presented in the recent literature [4, 18].

The UAM dataset uses only 10 electrodes that are already known to perform well in P300 recognition problem, but we can see from Figure 3 that the adaptive mixed-norm regularizer further selects some sensors that are essentially located in the occipital region. Note that despite the good average performances reported in Table 2, some subjects in this dataset achieve very poor performances, of about 50% of AUC, regardless of the considered method. Selected channels for one of these subjects (Subject 25) are depicted in Figure 3 and, interestingly, they strongly differ from those of other subjects providing rationales for the poor AUC.

**Figure 4:**
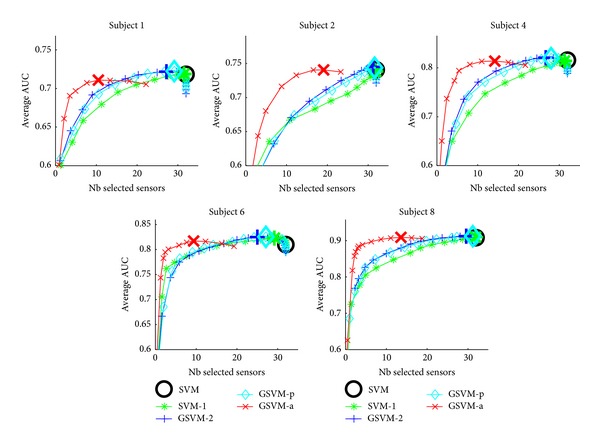
Performance versus sensor selection visualisation for the EPFL dataset. The large marker corresponds to the best model along the regularization path.

**Figure 5:**
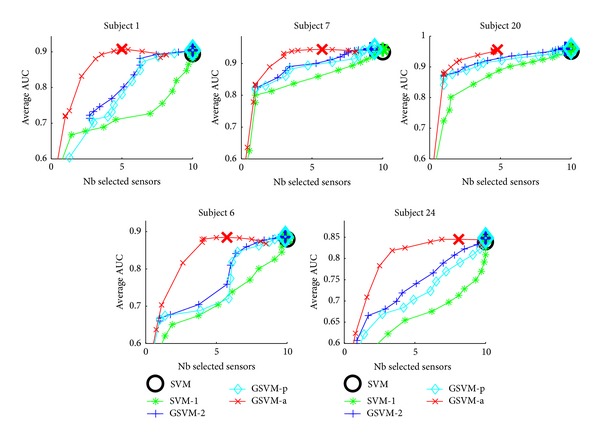
Performance versus sensor selection visualisation for the UAM dataset. The large marker corresponds to the best model along the regularization path.

We have also investigated the impact of sparsity on the overall performance of the classifiers. To this aim, we have plotted the average performance of the different classifiers as a function of the number of selected sensors. These plots are depicted in Figure 4 for the EPFL dataset and on Figure 5 for the UAM dataset. For both datasets, GSVM-a frequently achieves a better AUC for a given level of sparsity. For most of the subjects, GSVM-a performs as well as SVM but using far less sensors. A rationale may be that in addition to selecting the relevant sensors, GSVM-a may provide a better estimation of the classifier parameters leading to better performances for a fixed number of sensors. As a summary, we suggest thus the use of an adaptive mixed-norm regularizer instead of an *ℓ*_1_ − *ℓ*_2_ mixed-norm as in van Gerven et al. [19] when sparsity and channel selection are of primary importance.

#### 3.3.3. ErrP Dataset

**Figure 6:**
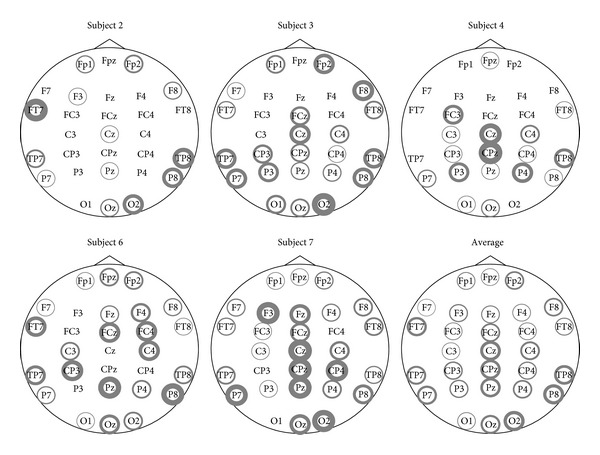
Selected sensors for the ERP dataset. The line width of the circle is proportional to the number of times the sensor is selected. No circle means that the sensor has never been selected.

The ErrP dataset differs from the others as its number of examples is small (72 examples per subject). The same experimental protocol as above has been used for evaluating the methods but only 57 examples out of 72 have been retained for validation/training. Classification performances are reported on Table 2. For this dataset, the best performance is achieved by GSVM-2 but the Wilcoxon test shows that all methods are actually statistically equivalent. Interestingly, many channels of this dataset seem to be irrelevant for the classification task. Indeed, GSVM-2 selects only 30% of them while GSVM-a uses only 7% of the channels at the cost of 10% AUC loss. We believe that this loss is essentially caused by the aggressive regularization of GSVM-a and the difficulty to select the regularization parameter *λ* using only a subset of the 57 training examples. Channels selected by GSVM-2 can be visualized on Figure 6. Despite the high variance in terms of selected sensors, probably due to the small number of examples, sensors in the central area seem to be the most selected one, which is consistent with previous results in ErrP [35].

#### 3.3.4. Multitask Learning

We now evaluate the impact of the approach we proposed in (10) and (11) on the P300 datasets. We expect that since multitask learning allows transferring some information between the different classification tasks, it will help in leveraging classification performances especially when the number of available training examples is small. Note that the ErrP dataset has not been tested in this MTL framework, because the above-described results suggest an important variance in the selected channels for all subjects. Hence, we believe that this learning problem does not fit into the prior knowledge considered through (11).

We have followed the same experimental protocol as for the single task learning except that training and test sets have been formed as follows. We first create training and test examples for a given subject by randomly splitting all examples of that subject and then gather all subject's training/test sets to form the multitask learning training/test sets. Hence, all the subjects are equally represented in these sets. A 3-fold nested cross-validation method is performed in order to automatically select the regularization terms (*λ*_*r*_ and *λ*_*s*_).

**Figure 7:**
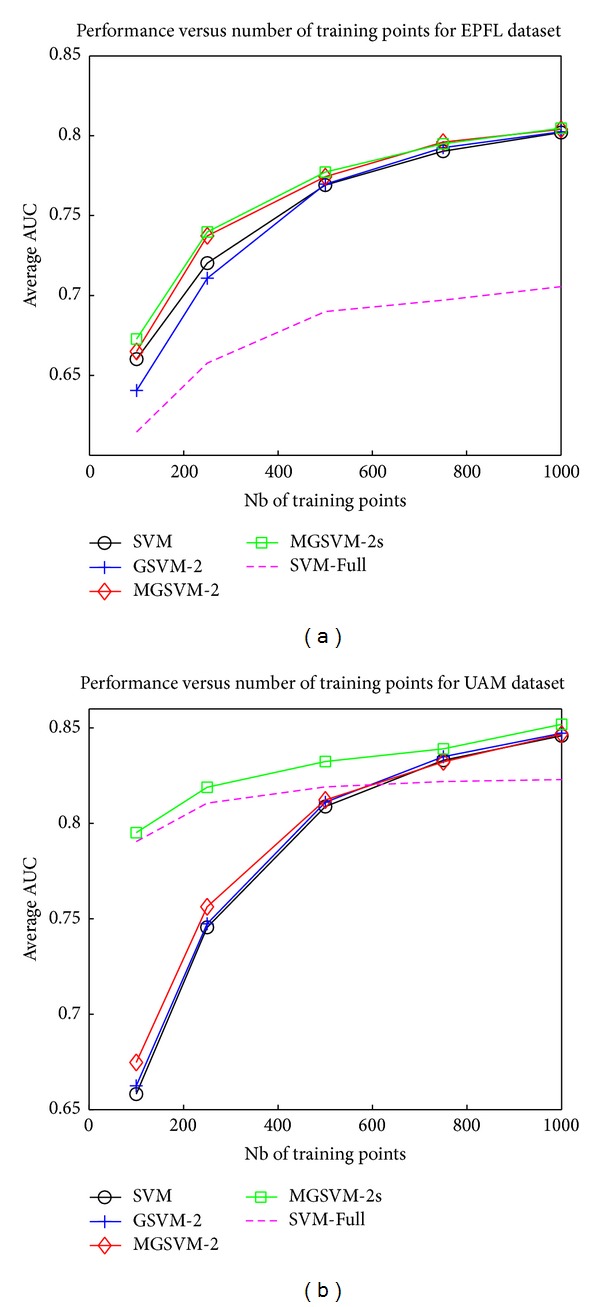
Multitask learning performances (AUC) for the EPFL (a) and UAM (b) datasets for different number of training examples per subject.

Performances of the different methods have been evaluated for increasing number of training examples per subject and are reported in Figure 7. We can first see that for the EPFL dataset, MGSVM-2 and MGSVM-2s yield a slight but consistent improvement over the single-task classifiers (SVM-Full being a single classifier trained on all subject's examples and SVM being the average performances of subject-specific classifiers). The poor performances of the SVM-Full approach are probably due to the high intersubject variability in this dataset, which includes impaired patients.

**Figure 8:**
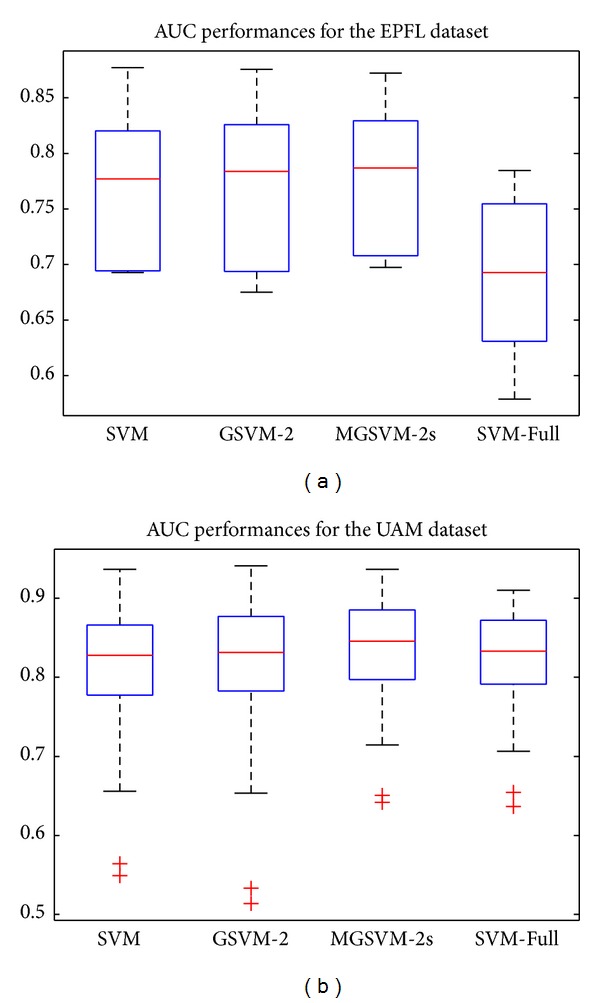
AUC performances comparison with EPFL (a) and UAM (b) for 500 training examples per subject.

For the UAM dataset, results are quite different since the SVM-Full and MGSVM-2s show a significant improvement over the single-task learning. We also note that when only the joint channel selection regularizer is in play (MGSVM-2), multitask learning leads to poorer performance than the SVM-Full for a number of trials lower than 500. We justify this by the difficulty of achieving appropriate channel selection based only on few training examples, as confirmed by the performance of GSVM-2. From Figure 8, we can see that the good performance of MGSVM-2s is the outcome of performance improvement of about 10% AUC over SVM, achieved on some subjects that perform poorly. More importantly, while performances of these subjects are significantly increased, those that perform well still achieve good AUC scores. In addition, we emphasize that these improvements are essentially due to the similarity-inducing regularizer.

For both datasets, the MTL approach MGSVM-2s is consistently better than those of other single-task approaches thanks to the regularization parameters *λ*_*r*_ and *λ*_*s*_ that can adapt to the intersubject similarity (weak similarity for EPFL and strong similarity for UAM). These are interesting results showing that multitask learning can be a way to handle the problem related to some subjects that achieve poor performances. Moreover, results also indicate that multitask learning is useful for drastically shortening the calibration time. For instance, for the UAM dataset, 80% AUC was achieved using only 100 training examples (less than 1 minute of training example recordings). Note that the validation procedure tends to maximize performances and does not lead to sparse classifiers for MTL approaches. As shown in Figures 2 and 3, the relevant sensors are quite different between subjects thus a joint sensor selection can lead to a slight loss of performances, hence the tendency of the cross-validation procedure to select nonsparse classifiers.

## 4. Conclusion

In this work, we have investigated the use of mixed-norm regularizers for discriminating event-related potentials in BCI. We have extended the discriminative framework of van Gerven et al. [19] by studying general mixed-norms and proposed the use of the adaptive mixed-norms as sparsity-inducing regularizers. This discriminative framework has been broadened to the multitask learning framework where classifiers related to different subjects are jointly trained. For this framework, we have introduced a novel regularizer that induces channel selection and classifier similarities. The different proposed approaches were tested on three different datasets involving a substantial number of subjects. Results from these experiments have highlighted that the *ℓ*_1_ − *ℓ*_2_ regularizer has been proven interesting for improving classification performance whereas adaptive mixed-norm is the regularizer to be considered when sensor selection is the primary objective. Regarding the multitask learning framework, our most interesting finding is that this learning framework allows, by learning more robust classifiers, significant performance improvement on some subjects that perform poorly in a single-task learning context.

In future work, we plan to investigate a different grouping of the features, such as temporal groups. This kind of group regularization could be for instance used in conjunction with the sensors group in order to promote both feature selection and temporal selection in the classifier. While the resulting problem is still convex, its resolution poses some issues so that a dedicated solver would be necessary.

Another research direction would be to investigate the use of asymmetrical MTL. This could prove handy when a poorly performing subject will negatively influence the other subject performances in MTL while improving his own performances. In this case one would like subject classifier to be similar to the other's classifier without impacting their classifiers.

## Figures and Tables

**Table 1:** Performance results on the simulated datasets: the average performance in AUC (in %), the average percent of selected sensors (Sel), and the *F*-measure of the selected channels (in %).

Methods	Avg. AUC	AUC *P*-val	Avg. Sel	*F*-measure
SVM	79.79	—	100.00	66.67
GSVM-1	79.32	0.027	98.75	67.25
GSVM-2	**80.96**	0.004	62.50	89.72
GSVM-p	80.74	0.020	63.12	89.40
GSVM-a	80.51	0.014	**45.62**	**93.98**

Best results for each performance measure are in bold.

The *P* value refers to the one of a Wilcoxon signed-rank test with SVM as a baseline.

**Table 2:** Performance results for the 3 datasets: the average performance (over subjects) in AUC (in %), the average percent of selected sensors (Sel), and the *P* value of the Wilcoxon signed-rank test for the AUC when compared to the baseline SVM's one.

Methods	Datasets								
	EPFL dataset (8 Sub., 32 Ch.)			UAM dataset (30 Sub., 10 Ch.)			ErrP dataset (8 Sub., 32 Ch)		
	Avg. AUC	Avg. Sel	*P* value	Avg. AUC	Avg. Sel	*P* value	Avg. AUC	Avg. Sel	*P* value
SVM	80.35	100.00	—	84.47	100.00	—	76.96	100.00	—
SVM-1	79.88	87.66	0.15	84.45	96.27	0.5577	68.84	45.85	0.3125
GSVM-2	**80.53**	78.24	0.31	**84.94**	88.77	0.0001	** 77.29**	29.84	0.5469
GSVM-p	80.38	77.81	0.74	**84.94**	90.80	0.0001	76.84	37.18	0.7422
GSVM-a	79.01	**26.60**	0.01	84.12	**45.07**	0.1109	67.25	**7.14**	0.1484

Best performing algorithms for each performance measure are in bold.

The *P* value refers to the one of a Wilcoxon signed-rank test with SVM as a baseline.

## Appendices

### A. Proof of Lipschitz Gradient of the Squared Hinge Loss

Given the training examples {**x**_*i*_, *y*_*i*_}, the squared Hinge loss is written as

(A.1) $$\begin{array}{c} J=\overset{n}{\underset{i=1}{\sum }}\underset{}{\max}{\left(0,1-y_{i}x_{i}^{T}w\right)}^{2}‍ \end{array}$$

and its gradient is

(A.2) $$\begin{array}{c} \nabla_{w}J=-2\underset{i}{\sum }x_{i}y_{i}\underset{}{\max}\left(0,1-y_{i}x_{i}^{T}w\right).‍\; \end{array}$$

The squared Hinge loss is gradient Lipschitz if there exists a constant *L* such that

(A.3) ||∇J(w1)−∇J(w2)||2≤L||w1−w2||2          ∀w1,w2∈ℝd.

The proof essentially relies on showing that **x**_*i*_ *y*_*i*_max⁡(0,1 − *y*_*i*_ **x**_*i*_^*T*^ **w**) is Lipschitz itself; that is, there exists *L*′ ∈ ℝ such that

(A.4) ||xiyimax⁡⁡(0,1−yixiTw1)−xiyimax⁡⁡(0,1−yixiTw2)|| ≤L′||w1−w2||.

Now let us consider different situations. For a given **w**_1_ and **w**_2_, if 1 − **x**_*i*_^*T*^ **w**_1_ ≤ 0 and 1 − **x**_*i*_^*T*^ **w**_2_ ≤ 0, then the left-hand side is equal to 0 and any *L*′ would satisfy the inequality. If 1 − **x**_*i*_^*T*^ **w**_1_ ≤ 0 and 1 − **x**_*i*_^*T*^ **w**_2_ ≥ 0, then the left-hand side (lhs) is

(A.5) lhs=||xi||2(1−xiTw2)≤||xi||2(xiTw1−xiTw2)≤||xi||22||w1−w2||2.

A similar reasoning yields to the same bound when 1 − **x**_*i*_^*T*^ **w**_1_ ≥ 0  1 − **x**_*i*_^*T*^ **w**_1_ ≤ 0 and 1 − **x**_*i*_^*T*^ **w**_2_ ≥ 0 and 1 − **x**_*i*_^*T*^ **w**_2_ ≥ 0. Thus, **x**_*i*_ *y*_*i*_max⁡(0,1 − *y*_*i*_ **x**_*i*_^*T*^ **w**) is Lipschitz with a constant ||**x**_*i*_||^2^. Now, we can conclude the proof by stating that ∇_**w**_ *J* is Lipschitz as it is a sum of Lipschitz function and the related constant is ∑_*i*=1_^*n*^||**x**_*i*_||_2_^2^.

### B. Lipschitz Gradient for the Multitask Learning Problem

For the multitask learning problem, we want to prove that the function

(B.1) ∑t=1m∑i=1nL(yi,t,xi,tTwt+bt)‍  ‍   +λs∑t=1m||wt−1m∑j=1mwj||22‍  

is gradient Lipschitz, *L*(·, ·) being the square Hinge loss. From the above results, it is easy to show that the first term is gradient Lipschitz as the sum of gradient Lipschitz functions.

Now, we also show that the similarity term

(B.2) $$\begin{array}{c} \underset{t}{\sum }{||w_{t}-\frac{1}{m}\overset{m}{\underset{j=1}{\sum }}w_{j}‍\;||}_{2}^{2} \end{array}$$

is also gradient Lipschitz.

This term can be expressed as

(B.3) ||wt−1m∑j=1mwj||22=∑t〈wt,wt〉‍−1m∑i,j=1m〈wi,wj〉‍  =wTMw,

where **w** ^*T*^ = [**w**_1_^*T*^,…, **w**_*m*_^*T*^] is the vector of all classifier parameters and **M** ∈ ℝ^*md*×*md*^ is the Hessian matrix of the similarity regularizer of the form

(B.4) $$\begin{array}{c} M=I-\frac{1}{m}\overset{m}{\underset{t=1}{\sum }}D_{t}‍ \end{array}$$

with **I** the identity matrix and **D**_*t*_ a block matrix with **D**_*t*_ a (*t* − 1)-diagonal matrix where each block is an identity matrix **I** with appropriate circular shift. **D**_*t*_ is thus a (*t* − 1) row-shifted version of **I**.

Once we have this formulation, we can use the fact that a function *f* is gradient Lipschitz of constant *L* if the largest eigenvalue of its Hessian is bounded by *L* on its domain [36]. Hence, since we have

(B.5) $$\begin{array}{c} {||M||}_{2}\leq {||I||}_{2}+\frac{1}{m}\overset{m}{\underset{t=1}{\sum }}{||D_{t}||}_{2}‍=2 \end{array}$$

the Hessian matrix of the similarity term 2 · **M** has consequently bounded eigenvalues. This concludes the proof that the function **w** ^*T*^ **M** **w** is gradient Lipschitz continuous.

### C. Proximal Operators

#### C.1. *ℓ*_1_ Norm

The proximal operator of the *ℓ*_1_ norm is defined as

(C.1) $$\begin{array}{c} \text{pro}{\text{x}}_{\lambda {||x||}_{1}}\left(u\right)=\underset{x}{\arg\;\min}\frac{1}{2}{||x-u||}_{2}^{2}+\lambda {||x||}_{1} \end{array}$$

and has the following closed-form solution for which each component is

(C.2) $$\begin{array}{c} {\left[\text{pro}{\text{x}}_{\lambda {||x||}_{1}}\left(u\right)\right]}_{i}=\mathrm{sign}\left(u_{i}\right){\left(\left|u_{i}\right|-\lambda \right)}_{+} \end{array}$$

#### C.2. *ℓ*_1_ − *ℓ*_2_ Norm

The proximal operator of the *ℓ*_1_ − *ℓ*_2_ norm is defined as

(C.3) proxλ∑g∈𝒢||xg||2(u) =arg min⁡x⁡12||x−u||22+λ∑g∈𝒢||xg||2.‍  

The minimization problem can be decomposed into several ones since the indices *g* are separable. Hence, we can just focus on the problem

(C.4) $$\begin{array}{c} \underset{x}{\min}\frac{1}{2}{||x-u||}_{2}^{2}+\lambda {||x||}_{2} \end{array}$$

whose minimizer is

(C.5) 0 if  ||u||2≤λ(1−λ||u||2)u otherwise.

## References

[1] Dornhege G, Millán J, Hinterberger T, McFarland D, Müller K (2007). *Toward Brain-Computer Interfacing*.

[2] Nunez PL, Srinivasan R (2006). *Electric Fields of the Brain*.

[3] Blankertz B, Müller K-R, Krusienski DJ (2006). The BCI competition III: validating alternative approaches to actual BCI problems. *IEEE Transactions on Neural Systems and Rehabilitation Engineering*.

[4] Rivet B, Cecotti H, Phlypo R, Bertrand O, Maby E, Mattout J EEG sensor selection by sparse spatial filtering in P300 speller Brain-Computer Interface.

[5] Blankertz B, Lemm S, Treder M, Haufe S, Müller K-R (2011). Single-trial analysis and classification of ERP components—a tutorial. *NeuroImage*.

[6] Müller-Gerking J, Pfurtscheller G, Flyvbjerg H (1999). Designing optimal spatial filters for single-trial EEG classification in a movement task. *Clinical Neurophysiology*.

[7] Gouy-Pailler C, Congedo M, Brunner C, Jutten C, Pfurtscheller G (2010). Nonstationary brain source separation for multiclass motor imagery. *IEEE Transactions on Bio-Medical Engineering*.

[8] Salimi-Khorshidi G, Nasrabadi AM, Golpayegani MH (2008). Fusion of classic P300 detection methods’ inferences in a framework of fuzzy labels. *Artificial Intelligence in Medicine*.

[9] Falkenstein M, Hohnsbein J, Hoormann J, Blanke L (1991). Effects of crossmodal divided attention on late ERP components—II. Error processing in choice reaction tasks. *Electroencephalography and Clinical Neurophysiology*.

[10] Ferrez P, Millán J (2007). *Error-Related Eeg Potentials in Brain-Computer Interfaces. Towards Brain-Computer Interfacing*.

[11] Buttfield A, Ferrez PW, Millán JDR (2006). Towards a robust BCI: error potentials and online learning. *IEEE Transactions on Neural Systems and Rehabilitation Engineering*.

[12] Krusienski DJ, Sellers EW, McFarland DJ, Vaughan TM, Wolpaw JR (2008). Toward enhanced P300 speller performance. *Journal of Neuroscience Methods*.

[13] Hoffman U, Yazdani A, Vesin J, Ebrahimi T Bayesian feature selection applied in a P300 brain—computer interface.

[14] Lal TN, Schröder M, Hinterberger T (2004). Support vector channel selection in BCI. *IEEE Transactions on Biomedical Engineering*.

[15] Yang J, Singh H, Hines EL (2012). Channel selection and classification of electroencephalogram signals: an artificial neural network and genetic algorithm-based approach. *Artificial Intelligence in Medicine*.

[16] Cecotti H, Rivet B, Congedo M (2011). A robust sensor-selection method for P300 brain-computer interfaces. *Journal of Neural Engineering*.

[17] Jrad N, Congedo M, Phlypo R (2011). Sw-SVm: sensor weighting support vector machines for EEG-based brain-computer interfaces. *Journal of Neural Engineering*.

[18] Rakotomamonjy A, Guigue V (2008). BCI competition III: dataset II-ensemble of SVMs for BCI P300 speller. *IEEE Transactions on Biomedical Engineering*.

[19] van Gerven M, Hesse C, Jensen O, Heskes T (2009). Interpreting single trial data using groupwise regularisation. *NeuroImage*.

[20] Tomioka R, Müller K-R (2010). A regularized discriminative framework for EEG analysis with application to brain-computer interface. *NeuroImage*.

[21] Evgeniou T, Pontil M Regularized multi-task learning.

[22] Argyriou A, Evgeniou T, Pontil M (2008). Convex multi-task feature learning. *Machine Learning*.

[23] Rakotomamonjy A, Flamary R, Gasso G, Canu S (2011). *ℓ*p*ℓ*q penalty for sparse linear and sparse multiple kernel multitask learning. *IEEE Transactions on Neural Networks*.

[24] Alamgir M, Grosse-Wentrup M, Altun Y (2010). Multi-task learning for brain-computer interfaces. *AI & Statistics*.

[25] Bach F, Jenatton R, Mairal J, Obozinski G, Sra S, Nowozin S, Wright SJ (2011). Convex optimization with sparsity-inducing norms. *Optimization for Machine Learning*.

[26] Rahimi A, Xu J, Wang L (2013). *L*
_*P*_-norm regularization in volumetric imaging of cardiac current sources. *Computational and Mathematical Methods in Medicine*.

[27] Liu A, Hao T, Gao Z, Su Y, Yang Z (2013). Non-negative mixed-norm convex optimization for mitotic cell detection in phase contrast microscopy. *Computational and Mathematical Methods in Medicine*.

[28] Bach FR (2008). Consistency of the group lasso and multiple kernel learning. *Journal of Machine Learning Research*.

[29] Zou H (2006). The adaptive lasso and its oracle properties. *Journal of the American Statistical Association*.

[30] Chapelle O (2007). Training a support vector machine in the primal. *Neural Computation*.

[31] Combettes P, Pesquet J (2011). Proximal splitting methods in signal processing. *Fixed-Point Algorithms for Inverse Problems in Science and Engineering*.

[32] Beck A, Teboulle M (2009). A fast iterative shrinkage-thresholding algorithm for linear inverse problems. *SIAM Journal on Imaging Sciences*.

[33] Hoffmann U, Vesin J-M, Ebrahimi T, Diserens K (2008). An efficient P300-based brain-computer interface for disabled subjects. *Journal of Neuroscience Methods*.

[34] Ledesma-Ramirez C, Bojorges Valdez E, Yáñez Suarez O, Saavedra C, Bougrain L, Gentiletti GG An open-access P300 speller database.

[35] Dehaene S, Posner M, Tucker D (1994). Localization of a neural system for error detection and compensation. *Psychological Science*.

[36] Bertsekas D (1999). *Nonlinear Programming*.

